# Risk factors for postoperative nausea and vomiting after general anesthesia: a systematic review and meta-analysis

**DOI:** 10.3389/fmed.2026.1791765

**Published:** 2026-03-18

**Authors:** Xiaoyan Dou, Xiuchun Yang, Yun Liu, Jiang Liu, Liuna Bi, Li Zhao, Fuchan Hu, Mengyao Huang, Jingyuan Zhang, Xun Zhou, Yan Jiang

**Affiliations:** 1 Weifang People’s Hospital, Weifang, China; 2 Yidu Central Hospital, Weifang, China; 3 Shandong Second Medical University, Weifang, China; 4 Xuzhou Medical University, Xuzhou, China

**Keywords:** PONV, postoperative complication, postoperative nausea and vomiting, predictor, risk factor

## Abstract

**Objective:**

This systematic review and meta-analysis aimed to quantitatively analyze and summarize the risk factors of postoperative nausea and vomiting (PONV) in patients undergoing general anesthesia.

**Methods:**

Literature screening, data extraction, and statistical analysis were conducted by an independent investigator. Meta-analyses were conducted for included studies using a random-effects method and generic inverse variance. All risk factors analyzed in the multifactorial regression models of the included studies were included in the meta-analysis to calculate the combined OR and 95% CI.

**Results:**

We included 31 studies (*n* = 355,117). Patient-specific factors included female gender (OR 2.39), motion sickness (2.08), non-smoking (1.70), migraine (1.38), BMI (1.06), age (0.98), ASA II (0.97) and ASA III (0.84). Anesthetic factors included patient-controlled analgesia (1.51), volatile anesthetics (1.46), postoperative-opioids (1.32), duration of anesthesia (1.31), intraoperative-opioids (1.22), N2O (1.12), intravenous-anesthesia (1.12), fentanyl (1.07), preventive antiemetic (0.84), and hormone medications (0.81). Of the 16 surgical factors, breast surgery (2.13), cholecystectomy (2.07), abdominal surgery (1.37), gynecology surgery (1.34), and surgical duration (1.13) were statistically significant. The remaining surgical factors, namely plastic (2.01), ophthalmologic surgery (2.0), neurosurgery (1.71), head/neck surgery (1.38), ear/nose/throat surgery (1.34), orthopedics (1.27), thyroid surgery (1.26), laparoscopic surgery (1.14), urological surgery (0.99), and postoperative pain (0.59) was not statistically significant.

**Conclusion:**

This study revealed patient-related, procedure-related, and anesthesia-related significant risk factors for PONV. This may provide a basis for clinical prevention, but a rigorously designed prospective study should be performed to confirm the findings of this study.

## Introduction

The incidence of postoperative nausea and vomiting (PONV) is up to 30–70% and constant over the last three decades. This can be attributed, at least in part, to the complexity of risk factors associated with PONV (1). PONV significantly influences the postoperative satisfaction of patients. Surveys on patient satisfaction after surgery indicate that PONV causes severe distress to patients (2, 3). Furthermore, it can also lead to serious complications such as postoperative bleeding, split sutures, fluid imbalance, and aspiration, thereby prolonging hospitalization and increasing healthcare costs (4–6). Although the risk-adapted strategy has been advocated in the past, the early risk prediction and control for PONV is supported according to recent trends in clinical guidelines. Risk prediction based on PONV risk factors has been proven to be beneficial for early prevention and control of PONV. Furthermore, when reasonable precautions are implemented based on patient and surgery-related risk-factors of PONV, it contributes to the hospital’s economic efficiency (7).

PONV is the result of the interaction of multiple risk factors. Currently identified risk factors may not be fully effective in the prevention of PONV (8). Different anesthetic techniques, surgical methods, and patient risk factors exert varying degrees of impact on the occurrence of PONV (9). Females, opioids, volatile anesthetics, history of PONV, non-smoking, and motion sickness have been identified as risk factors for PONV in previous studies (10, 11). Many more controversial risk factors for PONV have appeared in recent years (e.g., operation duration, migraine, anxiety) (12–14). Several systematic reviews revealed that the genetic factors are also associated with PONV (15, 16). The systematic analysis of PONV risk factors is helpful in identifying patients at high risk of PONV, thus further reducing the risk of PONV (17). Nonetheless, the findings of PONV risk factors in various studies are inconsistent. Previous systematic reviews of PONV risk factors mainly focused on the effect of a single factor on PONV (18–20). However, there is a lack of systematic evidence-based reviews quantifying the relative effects of independent risk factor of PONV, and failure to consider the influence of confounders. These contradictory findings and absence of systematic review and meta-analysis for multifactorial analyses of PONV emphasize the necessity of systematically analyzing individual risk factors of PONV. Multifactorial analysis is more likely to reflect the potential association, and can also be applied to explain or assess risks after correcting confounders.

Therefore, the purpose of this systematic review and meta-analysis is to quantitatively assess the impact of risk factors and potentially important confounders of PONV.

## Methods

### Protocol and registration

This systematic review was performed following the Meta-analysis of Observational Studies in Epidemiology (MOOSE) guidelines (21). The protocol of study was registered in the PROSPERO (CRD42023450016).

### Search strategy

A systematic search was conducted on PubMed, EMBASE, and Cochrane Library from inception until July 2025 to identify all available evidence. In order to identify all relevant trials, no restrictions were placed on the date, language, or status of publication. Reference lists of all studies satisfying eligibility criteria were screened for any potential studies. The clinical trial registry platform (www.clinicaltrials.gov) was also searched to identify potentially eligible ongoing or completed studies. The search strategy was composed using different combinations of the following Medical Subject Headings (MeSH terms) or related-keywords: ‘postoperative nausea and vomiting’, ‘PONV’, ‘Risk Factor’, ‘Risk Score’, ‘Predictor’, ‘Predictive model’, ‘Logistic Models’. The searching process was performed by an expert librarian.

### Eligibility criteria

Inclusion criteria: (1) any type of epidemiological observational trials (case–control studies or cohort study design), (2) investigating independent predictors associated with PONV by multifactorial logistic regression analysis, (3) participants of included studies were adult populations (>18 years) undergoing general anesthesia, (4) effect size data related to PONV risk factors were reported (odds ratios (ORs) or relative risk (RR), or able to be calculated from the data provided in the study). Only studies with more than 600 patients were included in this study. This study focuses on independent risk factors in multivariate logistic regression models. In logistic regression, small sample sizes may lead to model overfitting and unstable results. According to the statistical principle of Events Per Variable (EPV), each predictor requires at least 10 to 20 events to ensure validity (22). To guarantee sufficient statistical power and model stability, a sample size threshold of 600 was established based on the number of covariates and the incidence rate of PONV. For different studies with overlapping participant cohorts, the most recent and largest studies were included. Exclusion criteria: (1) studies for which the full text was not available even after contacting the authors, (2) studies with incomplete outcomes, (3) abstracts, case reports, animal trials and conference summary.

### Study selection

All retrieved literature were imported into EndNote© X9 for management. After removing duplicates, two independent researchers reviewed the titles and abstracts of the search results for selecting related studies. All potentially eligible articles were assessed in full text by another two investigators for inclusion in the systematic review. Any disagreements during the review process were resolved through consensus. Inter-rater reliability for study selection and data extraction was assessed using Cohen’s kappa coefficient (*κ*). The agreement between the two reviewers was strong (*κ* = 0.87).

### Assessment of risk of bias for individual trials

The methodological quality of individual studies was assessed by two independent reviewers using the Newcastle-Ottawa Scale (NOS). The NOS contains two different tools to assess case–control and cohort studies based on the selection of observation subjects, comparability between groups, and outcome measures, respectively (0–9, with a score of 0–5 indicating low quality and 6–9 indicating high quality). Differences in ratings were resolved by a joint re-assessment of the original studies. Quality score for each study was reported in Table 1.

**Table 1:** Characteristics of included studies.

Year, Author	Study design	Country	Center	Endpoint	%	Sample size	NOS
2022 Emi Ishikawa et al	Cohort study	Japan	1	PONV (0–24)	15.9	761	5
2021 Emma Johansson et al	Cohort study	Sweden	1	PONV (0–24)	9.6	1742	7
2020 Jong-Ho Kim et al	Cohort study	Korea	5	PONV (0–24)	10.5	187,706	8
2019 Jennifer R Majumdar et al	Cohort study	USA	1	PONV (0–24)	N/A	4,057	5
2019 Ulrike M Stamer et al	Cohort study	Switzerland	1	PONV (0–24)	51.6/68.2	918/1663	7
2021 Yan-Yuen Poon et al	Cohort study	China	1	PV (0–24)	13.1	665	6
2005 Jolanda E Van den Bosch et al	Cohort study	Netherlands	1	PONV (0–24)	48	1,389	8
2018 Myung Sub Yi et al	Cohort study	Korea	1	PONV (0–24)	18	6,773	7
2016 Paul S Myles et al	RCT	China	2	PONV (0–72)	12.4	7,011	7
2016 Florian Brettner et al	Cohort study	Germany	1	PONV (0–24)	12.9	2,617	7
2015 Y H Wu et al	Cohort study	China	1	PONV (0–24)	42	992	6
2020 Jinfei Li et al	Cohort study	China	1	PONV (0–24)	3.1	721	7
2012 Christian C Apfel et al	Cohort study	USA	12	PONV (0–2)	20.7	2,170	8
2012 Pankaj Sarin et al	Cohort study	USA	1	PONV (0–24)	N/A	2,505	7
2008 K Leslie et al	RCT	Australia	19	PONV (0–24)	16.6	2,500	8
1999 C C Apfel et al	Cohort study	Finland/ Germany	2	PONV (0–24)	55.6/31.3	520/2202	8
2007 Jan Wallenborn et al	Cohort study	Germany	1	PONV (0–24)	10.4	625	7
2023 Sirkku E Ahlström et al	Cohort study	Finland	1	PONV (0–24)	22.9	815	5
2023 Lili Qiu et al	Cohort study	China	1	PONV (0–6)	7.9	7,759	6
2021 Tomonori Morita et al	Cohort study	Japan	1	PONV (0–24)	24	928	5
2020 Wendy Suhre et al	Cohort study	USA	2	PONV (0–24)	17.5	27,388	7
2022 Jong-Ho Kim et al	Cohort study	Korea	1	PONV/PV (0–24)	12.9/2.6	60,656	6
1999 D R Sinclair et al	Cohort study	Canada	9	PONV (0–24)	9.1	17,638	8
1998 C C Apfel et al	Cohort study	Germany	1	PV (0–24)	36	1,091	6
2000 L H Eberhart et al	Cohort study	Germany	1	PONV (0–24)	37.4	1,444	7
2001 K Visser et al	RCT	Netherlands	1	PONV (0–72)	46	2010	7
2002 C C Apfel et al	Cohort study	Germany	1	PONV (0–24)	38.3	1,566	8
2003 Michaela Stadler et al	Cohort study	Belgium	1	PN/PV (0–72 h)	19/10	671	7
2004 C C Apfel et al	Cohort study	Germany	1	PONV (0–24)	38.3	1,566	8
2005 C C Apfel et al	Cohort study	International	28	PONV (0–24)	34	5,161	6
2008 Masashi Nakagawa et al	Cohort study	Japan	1	PONV (0–24)	14.9	1,070	7

### Data extraction

Data extraction for the included studies was conducted independently by an independent reviewer using an electronic data abstraction form and pilot tested with three articles. The extracted data would be cross checked by a second reviewer. The primary endpoint was PONV. PONV was defined as any degree of nausea/dry heaves with or without vomiting within 24 h after surgery. The primary metrics were all reported independent predictors of PONV, and pooled adjusted effect size data. From each eligible article, author, study area, year of publication, sample size, study design and basic participant characteristics (e.g., mean age) were also recorded.

### Data synthesis and statistical analysis

The primary endpoint in this study was the composite outcome of PONV. PONV is defined as nausea and/or vomiting occurring within 24 h postoperatively. We acknowledge that nausea and vomiting involve distinct physiological mechanisms. However, in clinical practice, prophylactic strategies typically target both symptoms simultaneously. Furthermore, the majority of included studies reported the composite endpoint derived from multivariate models. Data regarding isolated postoperative nausea (PN) or postoperative vomiting (PV) were insufficient to perform robust separate meta-analyses. Therefore, we pooled these outcomes to maximize statistical power and reflect real-world clinical endpoints. Meta-analyses were conducted for included studies using a random-effects method and generic inverse variance due to the unignorable heterogeneity between studies. Heterogeneity between studies was analyzed using Cochran’s *Q*-test and the *I*^2^-statistic. Sensitivity analyses were performed by excluding each study individually. To assess potential bias arising from differing study designs, we performed a sensitivity analysis by excluding the three randomized controlled trials (RCTs). All risk factors analyzed in the multifactorial regression models of the included studies, including factors that were not statistically significant, were included in the meta-analysis to calculate the combined OR and 95% confidence intervals (CI). Adjusted odds ratios (ORs) from the included studies were extracted directly from the multivariate models of primary studies to account for identified confounding factors. A random-effects model was employed to address potential heterogeneity arising from differing sets of covariates across the included studies. For meta-analyses that included more than 10 studies, egger test was used to assess the potential publication bias. We acknowledge that the egger test may lack statistical power to detect publication bias, even when more than 10 studies are included. Meta-regression analysis was used to explore possible sources of heterogeneity. NOS score, number of research centers, country, and study year were considered as covariates in the meta-regression analysis. All statistical analyses were performed using STATA (version 16.0). *p*-values < 0.05 were considered as statistically significant.

## Results

### Study selection

**Figure 1:**
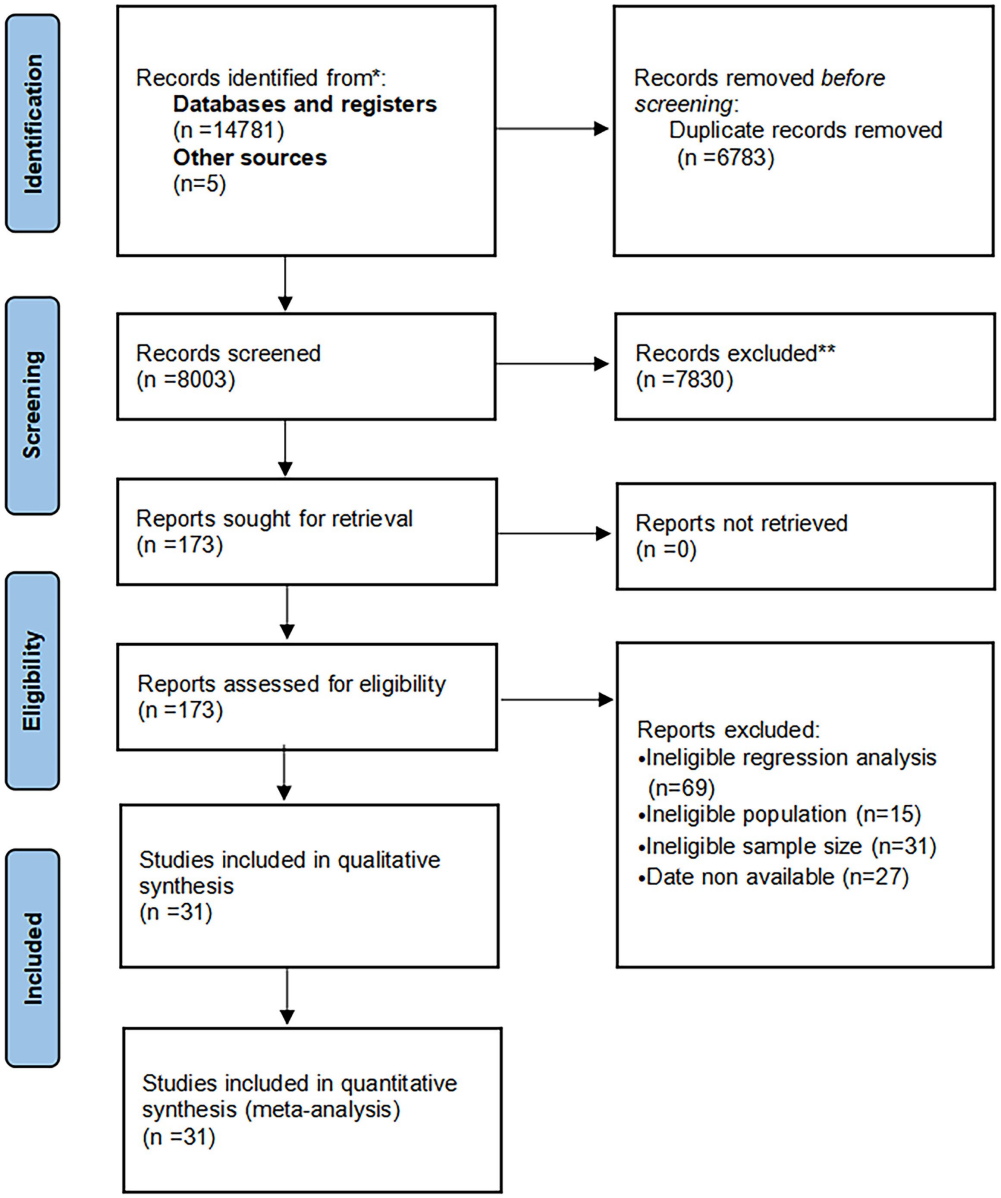
PRISMA flow diagram of this meta-analysis.

A total of 14,786 studies were identified in the initial screening. After removing 6,783 duplicates, 173 studies were screened as eligible records based on title and abstract. A total of 142 studies were excluded after full-text review for the following reasons: 69 studies did not use multifactorial logistic regression to analyze independent risk factors, 31 studies included <600 populations, 15 studies enrolled children aged <18 yr., and 27 studies contained no relevant outcomes or reported incomplete outcomes. Ultimately, 31 studies met the eligibility criteria of this study and were included in the systematic review and meta-analysis (8, 12, 13, 23–50). The process of study screening is shown in Figure 1.

### Study characteristics

The 31 studies involving 355,117 participants were included in this systematic review. Of these, four studies reported data for PN and PV, and another five studies reported PONV as the study endpoint. Overall, the median incidence of PN/PV or PONV as the primary endpoint was 17.8 (2.6–68.2). The 31 included studies involved 8 patient-specific factors, 16 surgical factors, and 10 anesthetic factors. For the study design, there were 3 RCTs and 28 cohort studies. Based on the study patients, the average age of the participants varied from 18 to 85. The characteristics of the included studies are presented in Table 1.

### Quality assessment

The NOS scores of 27 studies were greater than or equal to 6 and were judged to be of high quality. The remaining studies were evaluated as low quality (NOS < 6). The assessment results of the study quality are detailed in Table 1.

### Pooled risk estimates of PONV

**Figure 2:**
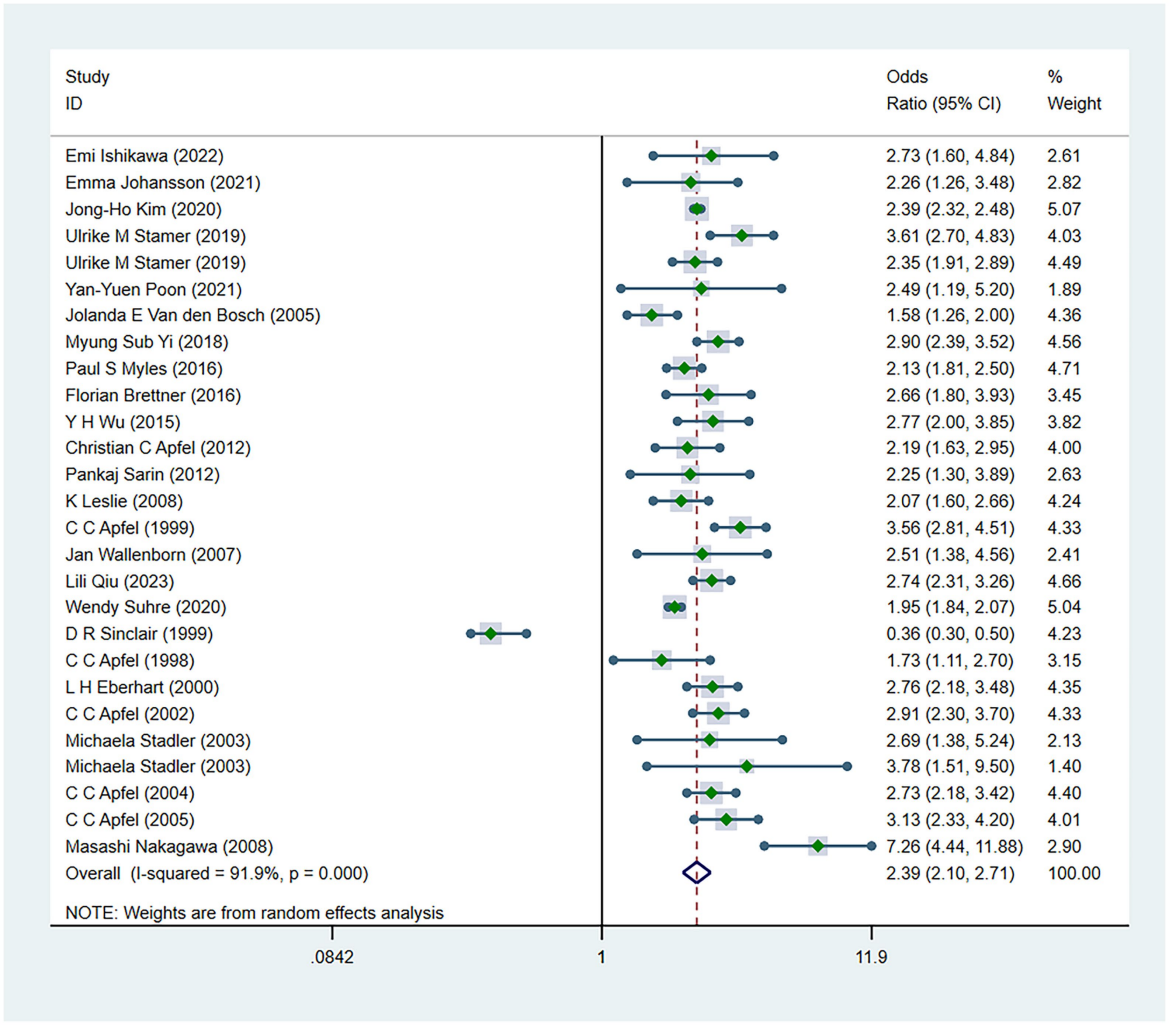
Forest plot displaying the results of ORs and 95% CIs for the risk factors of female gender.

**Figure 3:**
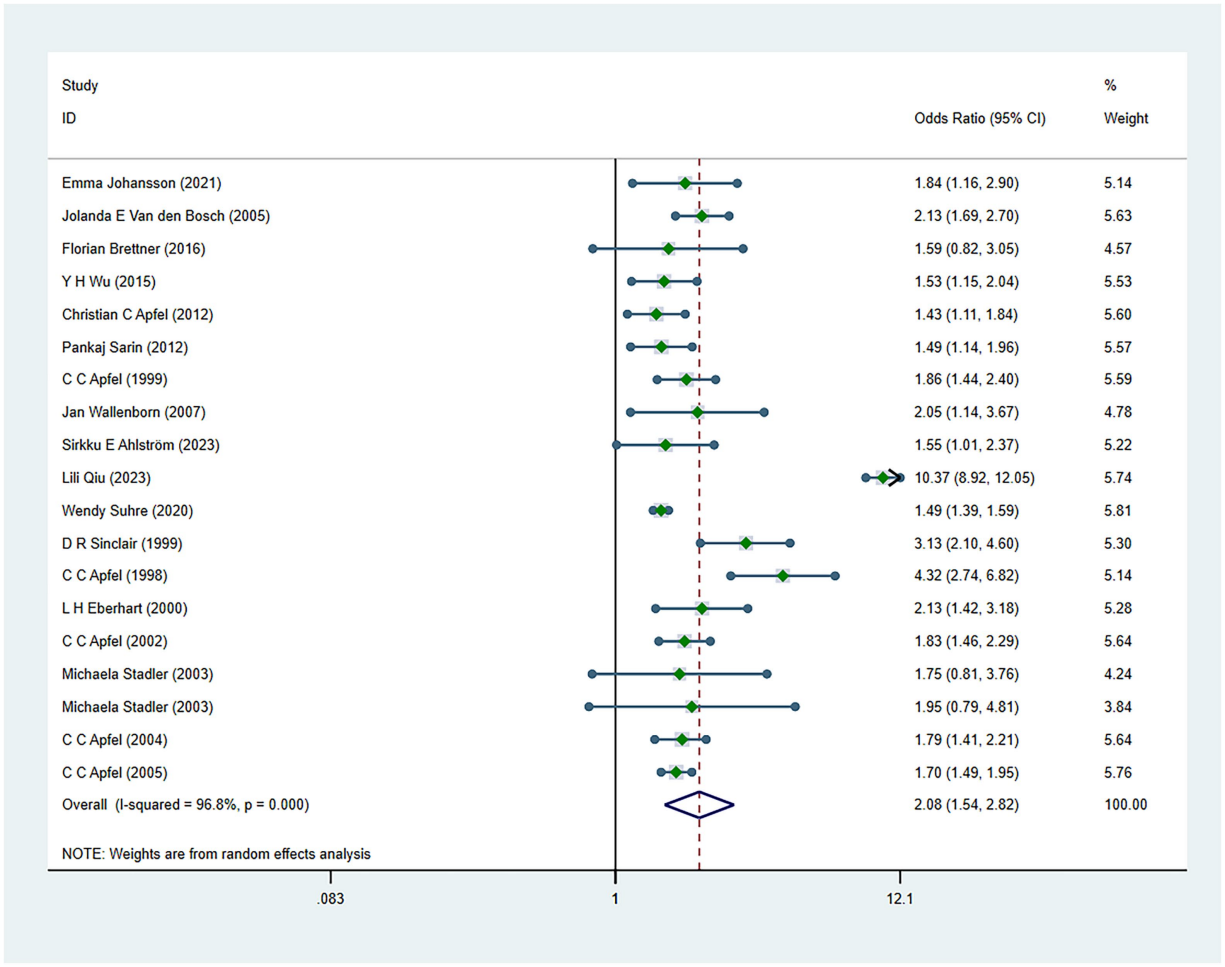
Forest plot displaying the results of ORs and 95% CIs for the risk factors of motion sickness.

**Figure 4:**
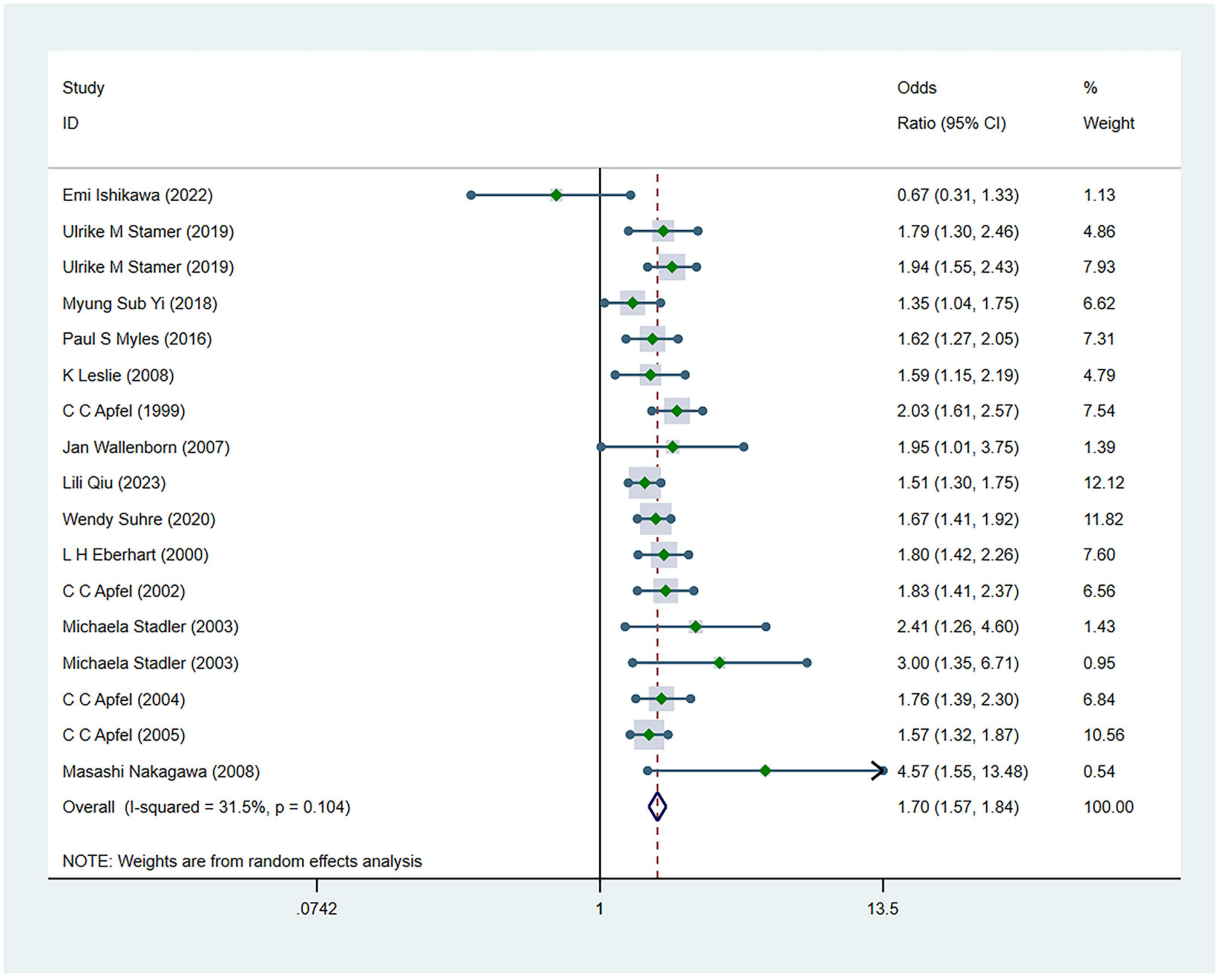
Forest plot displaying the results of ORs and 95% CIs for the risk factors of smoking.

**Figure 5:**
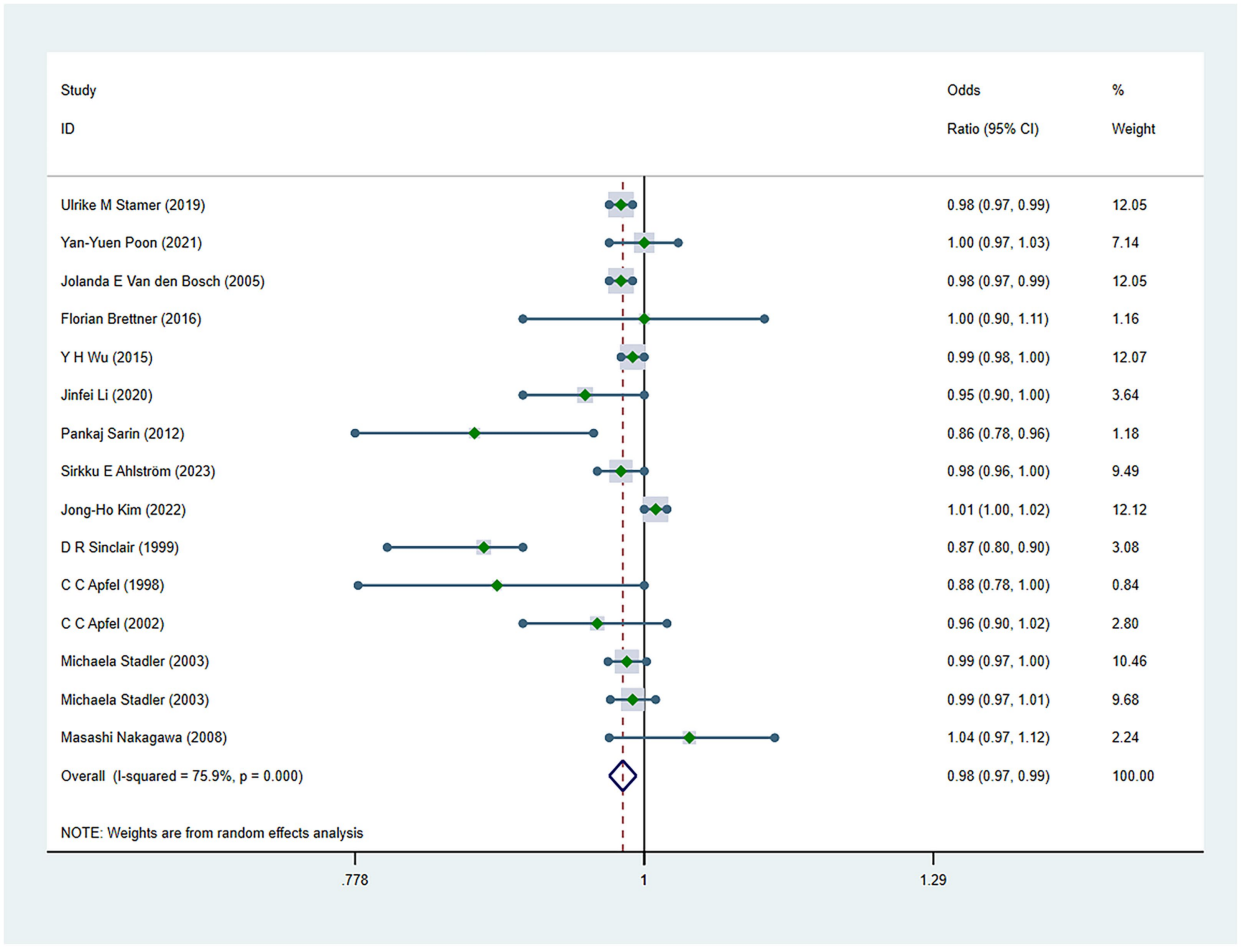
Forest plot displaying the results of ORs and 95% CIs for the risk factors of age.

**Figure 6:**
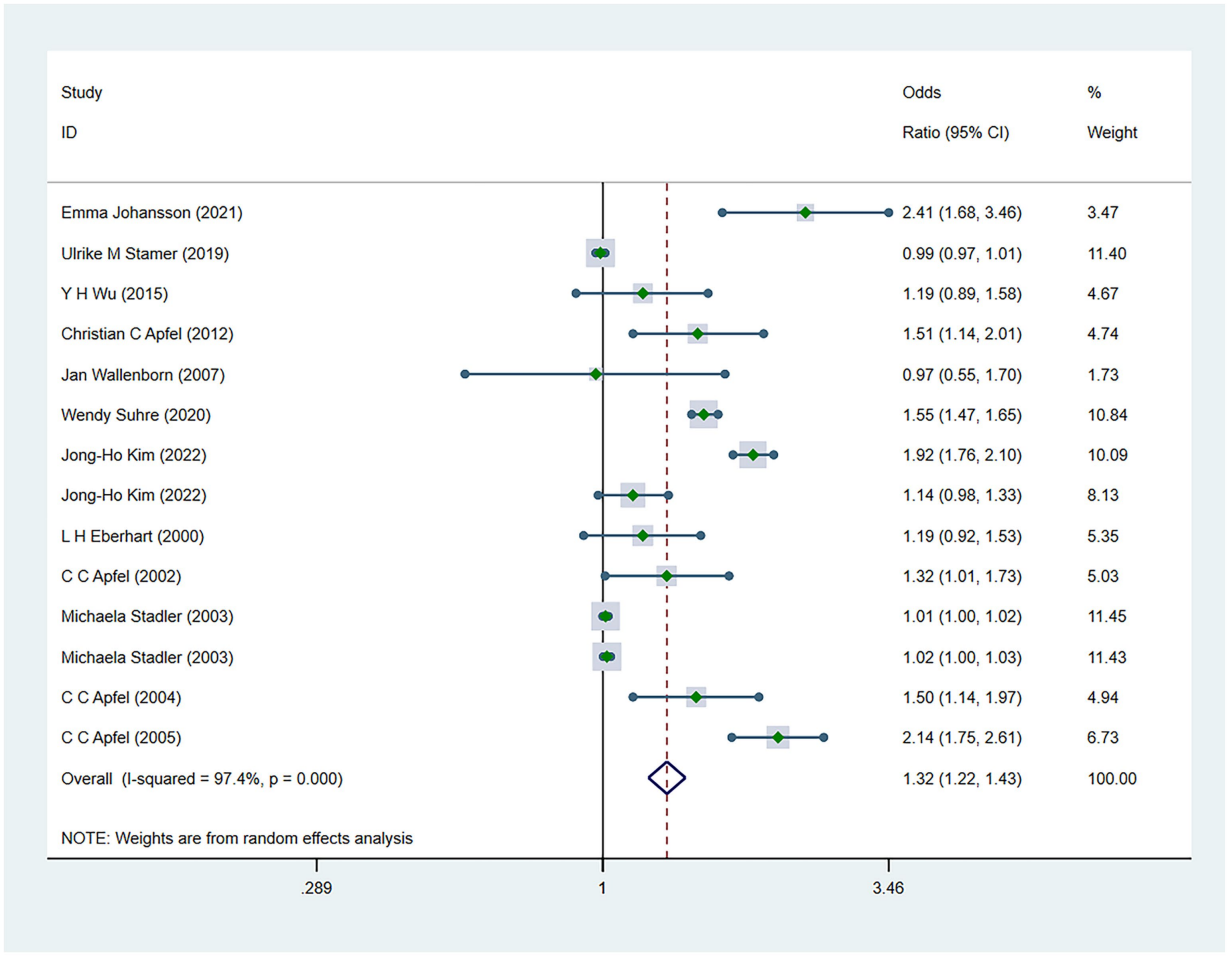
Forest plot displaying the results of ORs and 95% CIs for the risk factors of postoperative opioids.

There were 34 risk factors included in the meta-analysis, including 8 patient-related factors, 10 anesthesia-related factors, and 16 surgery-related factors independently associated with PONV (Table 2). Patient-specific factors included female gender (OR, 2.39; *I*^2^ = 91.9%) (Figure 2), motion sickness (OR, 2.08; *I*^2^ = 96.8%) (Figure 3), non-smoking (OR, 1.70; *I*^2^ = 31.5%) (Figure 4), migraine (OR, 1.38; *I*^2^ = 0%), BMI (OR, 1.06; *I*^2^ = 96.9%), age (OR, 0.98; *I*^2^ = 75.9%) (Figure 5), ASA II (OR, 0.97; *I*^2^ = 73.2%) and ASA III (OR, 0.84; *I*^2^ = 66.6%). Anesthetic factors included patient-controlled analgesia (OR, 1.51; *I*^2^ = 64.3%), volatile anesthetics (OR, 1.46; *I*^2^ = 92.5%), postoperative-opioids (OR, 1.32; *I*^2^ = 97.4%) (Figure 6), duration of anesthesia (OR, 1.31; *I*^2^ = 93.7%), intraoperative-opioids (OR, 1.22; *I*^2^ = 77.4%), N2O (OR1.12; *I*^2^ = 96.6%), intravenous-anesthesia (OR, 1.12; *I*^2^ = 91.2%), fentanyl (OR, 1.07; *I*^2^ = 63.7%), preventive antiemetic (OR, 0.84; *I*^2^ = 97.6%), and hormone-based medications (OR, 0.81; *I*^2^ = 84.6%). Of the 16 surgical factors, breast surgery (OR, 2.13; *I*^2^ = 38.6%), cholecystectomy (OR, 2.07; *I*^2^ = 71.8%), abdominal surgery (OR, 1.37; *I*^2^ = 50.1%), gynecology surgery (OR, 1.34; *I*^2^ = 88.6%), and surgical duration (OR, 1.13; *I*^2^ = 98.2%) were statistically significant. The remaining surgical factors, namely plastic (OR, 2.01; *I*^2^ = 90.0%), ophthalmologic surgery (OR, 2.0; *I*^2^ = 86.2%), neurosurgery (OR, 1.71; *I*^2^ = 93.7%), head/neck surgery (OR, 1.38; *I*^2^ = 14.8%), ear/nose/throat surgery (OR, 1.34; *I*^2^ = 80.5%), orthopedics (OR, 1.27; *I*^2^ = 91.0%), thyroid surgery (OR, 1.26; *I*^2^ = 84.7%), laparoscopic surgery (OR, 1.14; *I*^2^ = 94.3%), urological surgery (OR, 0.99; *I*^2^ = 76.0%), and postoperative pain (OR, 0.59; *I*^2^ = 69.4%) were not statistically significant. Regarding fasting time, only one study reported that long fasting time would increase the risk of PONV. The variation in effect sizes and heterogeneity for all pooled results is illustrated in Table 3.

**Table 2:** ORs of risk factors associated with PONV in included studies.

Study	Patient-related risk factors								Anesthesia-related risk factors									
	Female gender	Age (>55)	Non-smoking	MS	BMI	Migrain	ASA (II)	ASA (III)	Duration	Volatile-anesthetics	N2O	Intravenous-anesthesia	PCA	Opioids-intraoperative	Opioids-postoperative	Ondansetron	Fentanyl	Hormone
Emi Ishikawa	2.73		0.67		1.0												1.06	
Emma Johansson	2.26			1.84	2.1				2.1					2.2	2.41	0.29		
Jong-Ho Kim	2.39					1.37		0.87		1.14	0.79		2.13	1.22		1.37		
Jennifer R Majumdar																		
Ulrike M Stamer	3.61 2.35	– 0.98	1.79 1.94												0.99 –	0.85 1.34	0.93 1.05	
Yan-Yuen Poon	2.49	1.0						0.74					0.73					
Jolanda E Van den Bosch	1.58	0.98		2.13						2.05								
Myung Sub Yi	2.9		1.35							2.21	1.54			1.3				
Paul S Myles	2.13		1.62					1.05						1.01				
Florian Brettner	2.66	1.0		1.59	1.08		0.56	0.41										
Y H Wu	2.77	0.99		1.53											1.19			
Jinfei Li		0.95												3.93		1.01		
Christian C Apfel	2.19			1.43											1.51	0.7	1.48	
Pankaj Sarin	2.25	0.86		1.49						0.72	1.86					0.71		0.71
K Leslie	2.07		1.59						1.3		2.04							
C C Apfel	3.56		2.03	1.86														
Jan Wallenborn	2.51		1.95	2.05	1.02				1.36	1.1					0.97			1.22
Sirkku E Ahlström		0.98		1.55			0.59	0.43										
Lili Qiu	2.74		1.51	10.37	1.38													
Tomonori Morita																		
Wendy Suhre	1.95		1.67	1.49			1.02	0.93		1.81	1.12				1.55	0.87		
Jong-Ho Kim		– 1.01			– 0.98		1.0 1.4			1.66 1.17	0.66 0.87	0.48 0.79			1.92 1.14			0.89 0.99
D R Sinclair	0.36	0.87		3.13					1.59									
C C Apfel	1.73	0.88		4.32														
L H Eberhart	2.76		1.80	2.13					1.82						1.19			
K Visser										2.03								
C C Apfel	2.91	0.96	1.83	1.83	0.82				1.36					1.86	1.32			
Michaela Stadler	2.69 3.78	0.99 0.99	2.41 3.0	1.75 1.95	1.0 0.94	2.15 1.27			0.93 0.72			2.51 3.67			1.01 1.02			
C C Apfel	2.73		1.76	1.79											1.5			
C C Apfel	3.13		1.57	1.70					1.2					0.96	2.14	0.56		0.57
Masashi Nakagawa	7.26	1.04	4.57						0.99									

Study	Surgery-related factors															
	Duration	Laparo-scopic	Chole-cyst	Gynae-cology	ENT	Thyroid	Plastic	Orth-opedics	Urology	Neurology	Head/Neck	Breast	Abdominal	Ophthal-mology	Pain	Fasting-time
Emi Ishikawa	0.999							5.69								
Emma Johansson		0.49														
Jong-Ho Kim		1.05														
Jennifer R Majumdar	1.13															
Ulrike M Stamer		1.26 1.24														
Yan-Yuen Poon	0.69															
Jolanda E Van den Bosch																
Myung Sub Yi																
Paul S Myles		0.74		0.74	0.43		0.52	0.34	0.6	0.32						
Florian Brettner				0.73					0.71			0.4				
Y H Wu		1.69				0.68		0.97			1.72	1.15				
Jinfei Li																
Christian C Apfel	1.83	2.39	2.8	1.5	1.17				0.34			0.82	0.97			
Pankaj Sarin	1.2		2.32	1.96									1.78		1.13	
K Leslie																
C C Apfel	1.79															
Jan Wallenborn																
Sirkku E Ahlström																
Lili Qiu	1.017	2.06														
Tomonori Morita																
Wendy Suhre	1.43															
Jong-Ho Kim	1.09 1.15	0.93 0.82	1.54 2.26	1.17 0.77												1.01 1.01
D R Sinclair				3.31	4.39		6.68	2.82						5.85		
C C Apfel		0.36				2.71		0.92				2.24	0.89	0.69		
L H Eberhart																
K Visser																
C C Apfel		1.63	2.85	1.11	1.0			1.37					1.23	1.36		
Michaela Stadler				9.3 0.84	3.35 1.51		2.94 1.7	2.72 0.98	8.09 6.17	4.81 0.99	5.0 1.77		5.75 1.21	9.49 2.14	0.37 0.27	
C C Apfel			3.23	1.84	1.6			1.3					2.0	1.45		
C C Apfel			1.49	1.78		1.22		0.91			1.08	0.74	1.04			
Masashi Nakagawa										6.41						

MS, motion sickness. ENT, ear, nose and throat. PCA, patient-controlled analgesia.

**Table 3:** Combined assessment of risk factors for PONV.

Risk factors	Study (*n*)	Participants (*n*)	Combined OR (95% CI)	*p*-value	Heterogeneity
Patient-related factors					
Female gender	25	288,264	2.39 (2.10 to 2.71)	<0.001	91.9
Age (>55)	14	94,730	0.98 (0.97 to 0.99)	0.002	75.9
Non-smoking	15	69,749	1.70 (1.57 to 1.84)	<0.001	31.5
Motion sickness	18	80,012	2.08 (1.54 to 2.82)	<0.001	96.8
BMI	8	77,068	1.06 (0.95 to 1.18)	0.300	96.9
Migrain	2	189,048	1.38 (1.22 to 1.57)	<0.001	0
ASA (II)	4	152,132	0.97 (0.78 to 1.19)	0.744	73.2
ASA (III)	6	226,202	0.84 (0.73 to 0.98)	<0.029	66.6
Anesthesia-related factors					
Duration	8	32,018	1.31 (1.12 to 1.53)	0.001	93.7
Volatile-anaesthetics	8	349,708	1.46 (1.19 to 1.80)	<0.001	92.5
N2O	6	348,184	1.12 (0.89 to 1.41)	0.327	96.6
Intravenous-anesthesia	2	122,654	1.12 (0.59 to 2.12)	0.738	91.2
PCA	2	188,371	1.51 (0.57 to 4.02)	0.412	64.3
Opioids (Intraoperative)	7	210,680	1.22 (1.03 to 1.44)	0.02	77.4
Opioids (Postoperative)	12	166,226	1.32 (1.22 to 1.43)	<0.001	97.4
Preventive antiemetic	8	229,974	0.84 (0.72 to 0.97)	0.015	97.6
Fentanyl	3	5,512	1.07 (0.96 to 1.20)	0.223	63.7
Hormone medications	4	129,608	0.81 (0.62 to 1.05)	0.116	84.6
Surgery-related factors					
Duration	9	168,819	1.13 (1.10 to 1.16)	<0.001	98.2
Laparoscopic	10	333,930	1.14 (0.94 to 1.38)	0.171	94.3
Cholecystectomy	6	134,280	2.07 (1.63 to 2.62)	<0.001	71.8
Gynecology	10	162,888	1.34 (1.04 to 1.73)	0.026	88.6
Ear, Nose and Throat	6	31,293	1.34 (0.75 to 2.36)	0.333	80.5
Thyroid	3	7,244	1.26 (0.65 to 2.44)	0.503	84.7
Plastic	3	25,991	2.01 (0.42 to 9.72)	0.384	90.0
Orthopedics	9	37,128	1.27 (0.76 to 2.10)	0.364	91.0
Urology	4	13,140	0.99 (0.46 to 2.14)	0.981	76.0
Neurology	3	9,423	1.71 (0.25 to 11.54)	0.584	93.7
Head/Neck	3	7,495	1.38 (0.85 to 2.26)	0.192	14.8
Breast	5	12,031	2.13 (1.62 to 2.80)	<0.001	38.6
Abdominal	7	15,396	1.37 (1.05 to 1.79)	0.021	50.1
Ophthalmology	5	23,203	2.00 (0.85 to 4.69)	0.111	86.2
Pain	2	3,847	0.59 (0.22 to 1.62)	0.308	69.4
Fasting time	1	121,312	1.01 (1.005 to 1.015)	<0.001	0

### Sensitivity analysis

**Figure 7:**
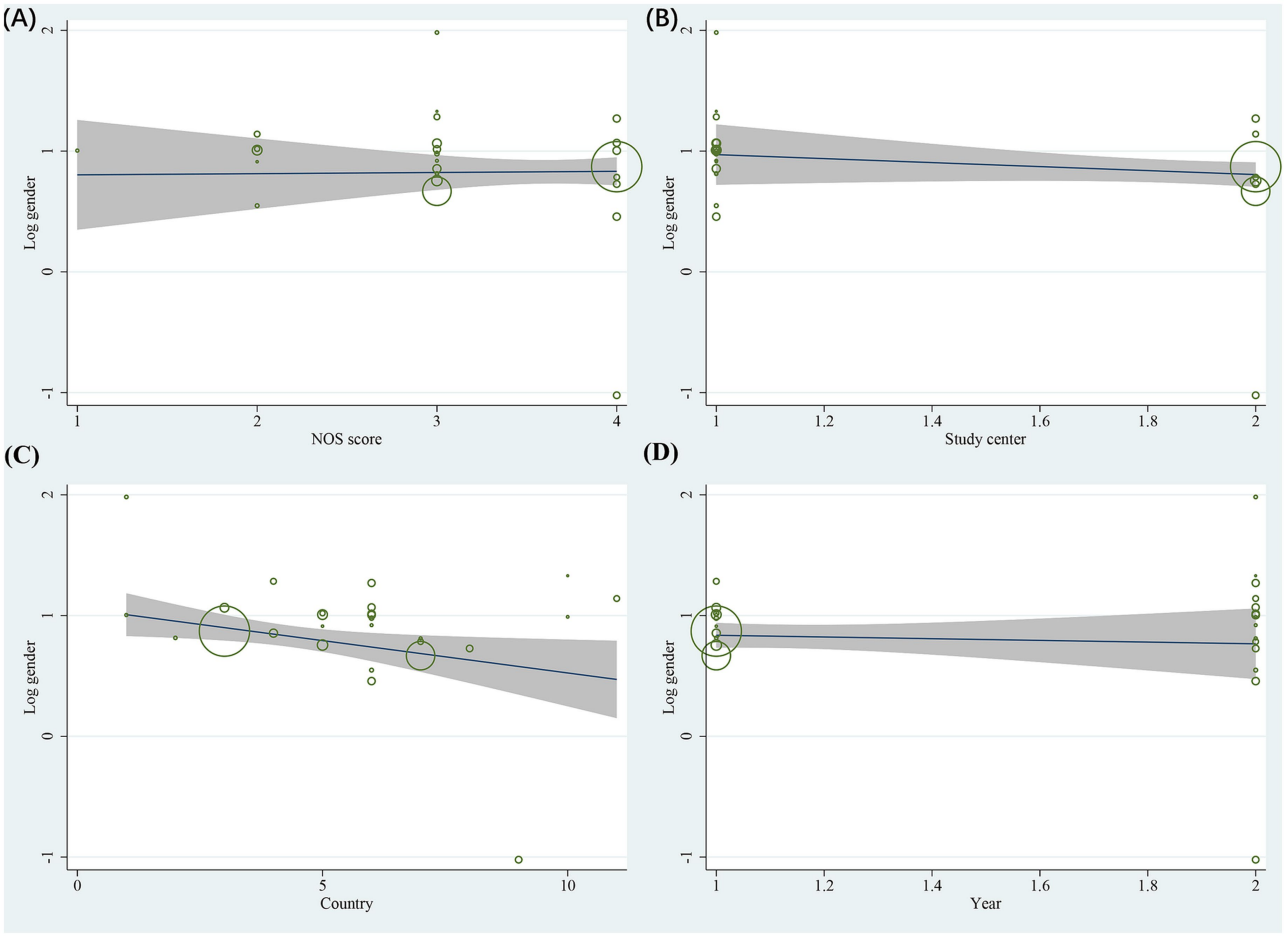
Results of meta-regression analysis for gender factor.

**Figure 8:**
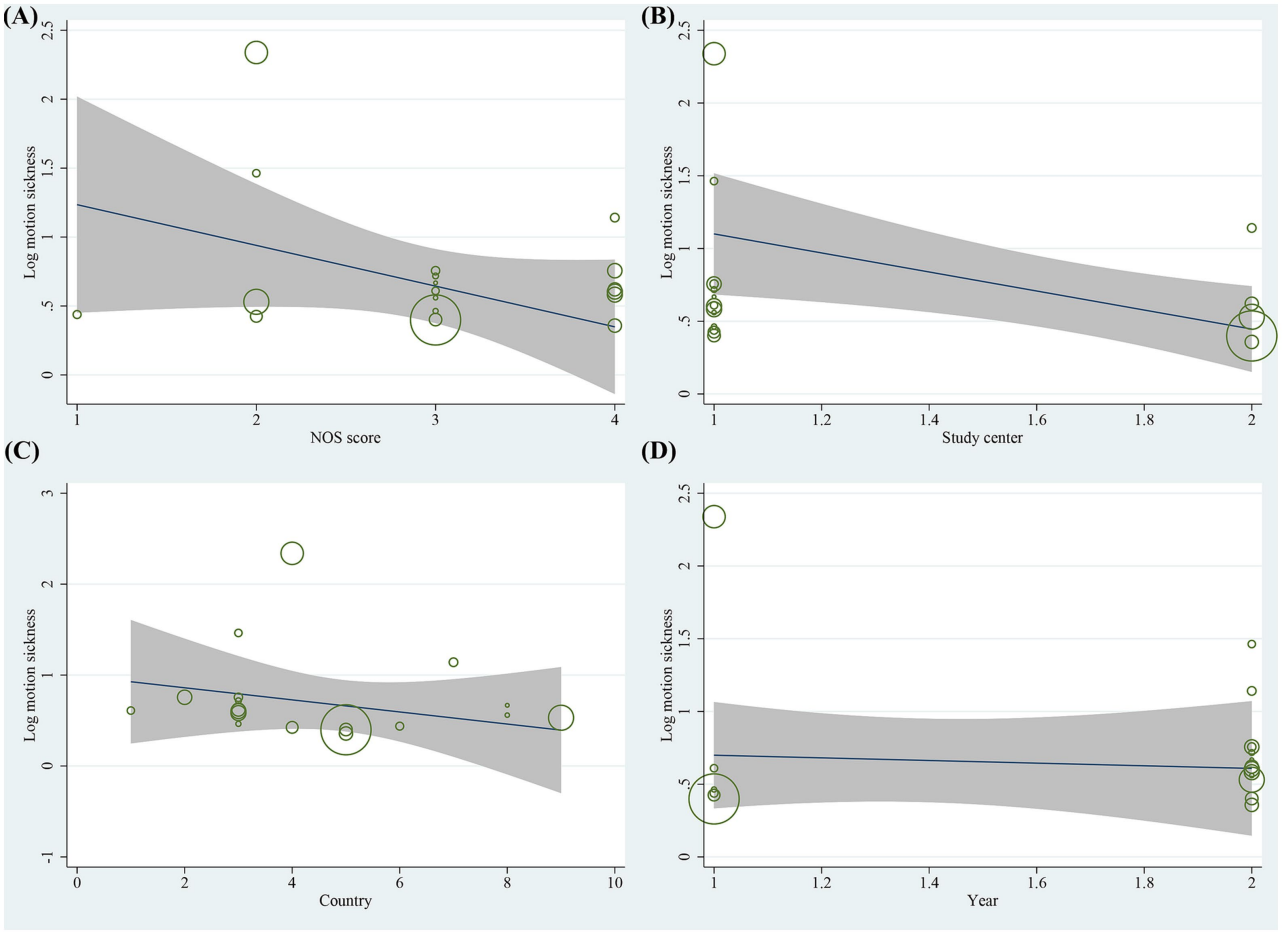
Results of meta-regression analysis for motion sickness factor.

**Figure 9:**
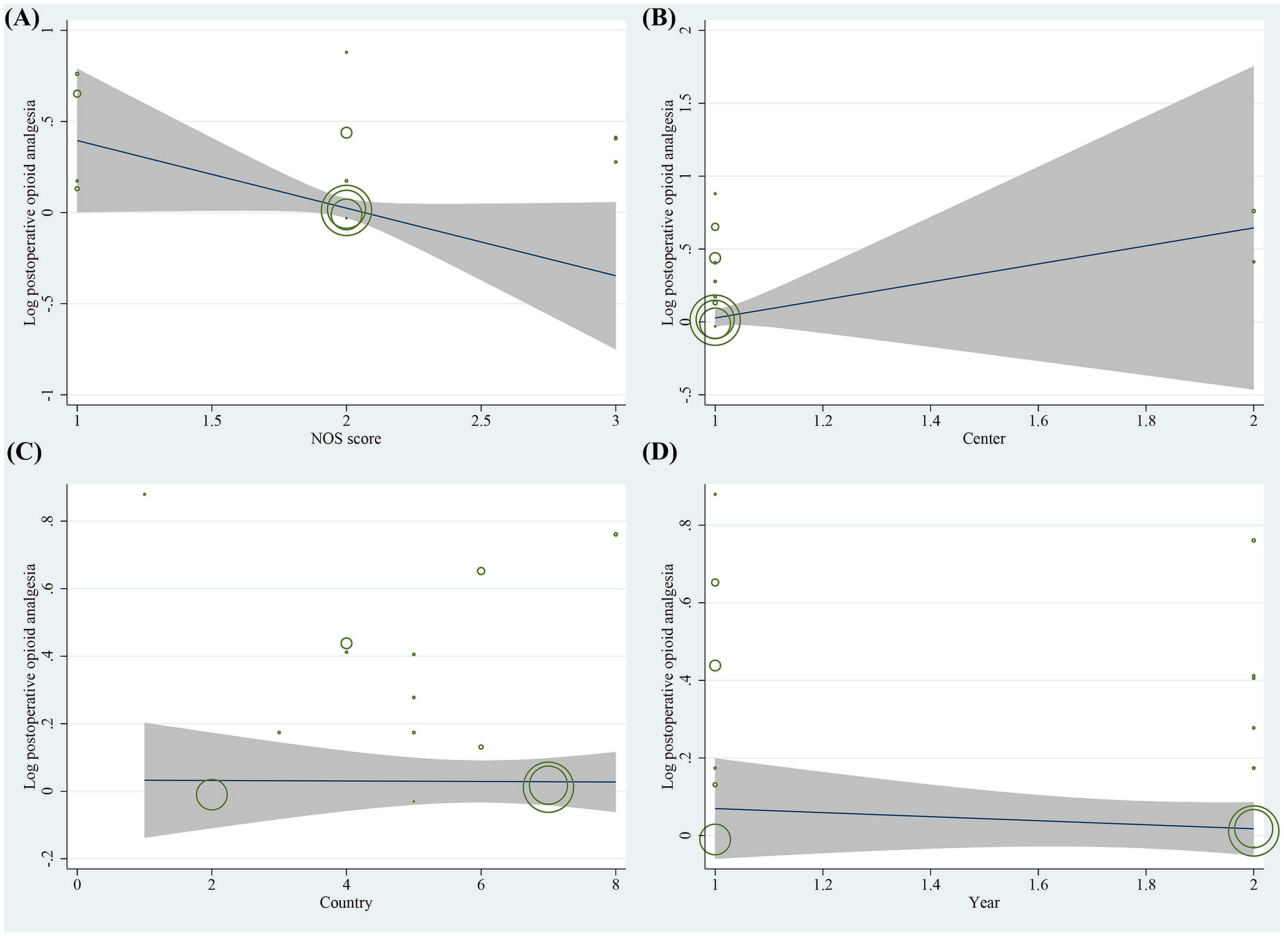
Results of meta-regression analysis for postoperative opioids factor.

**Figure 10:**
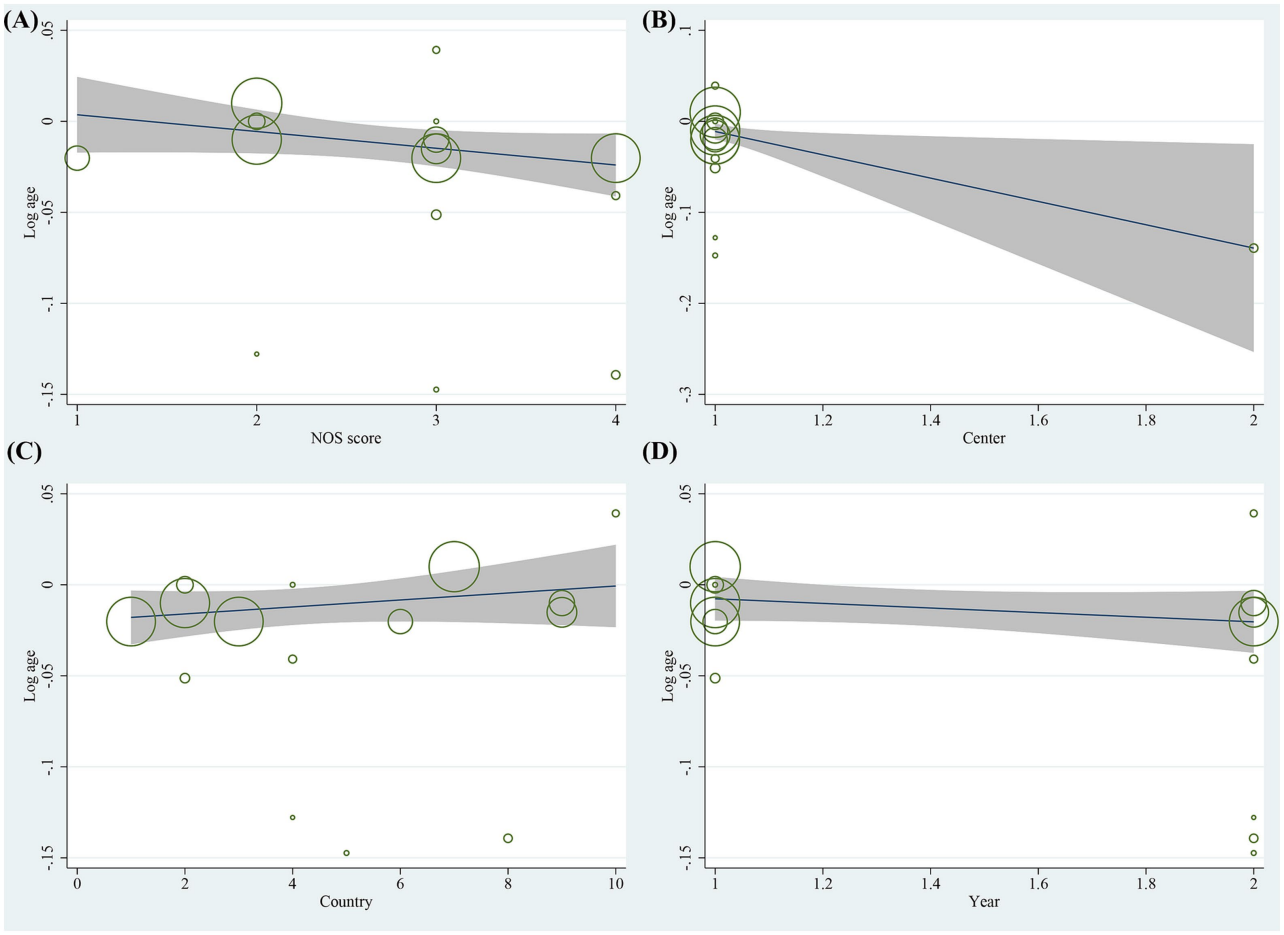
Results of meta-regression analysis for age factor.

Sensitivity analyses of the included studies revealed no significant variation in the pooled effect sizes and heterogeneity after excluding each study one by one. This suggests that all studies contributed almost equally to the results of the meta-analysis. Sensitivity analyses excluding the three RCTs demonstrated that the pooled ORs for primary risk factors (e.g., female gender, motion sickness, non-smoking) remained consistent with the primary analysis. The magnitude and statistical significance of the associations were unchanged. This suggests that the inclusion of RCT data did not introduce substantial bias to the overall conclusions. Heterogeneity tests indicated that an individual study was not the significant source of heterogeneity. Meta-regression analysis was performed to explore sources of heterogeneity. For the gender factor, covariates including NOS score (*p* = 0.220), study center (*p* = 0.092), country (*p* = 0.067), and study year (*p* = 0.631) were not significant sources of heterogeneity (Figure 7). Similarly, for motion sickness and postoperative opioids, no significant sources of heterogeneity were identified among these covariates (Figures 8, 9). However, for the age factor, the number of study centers (*p* = 0.007) was identified as a significant source of heterogeneity (Figure 10).

### Publications bias

The results of egger test revealed that motion sickness (*p* = 0.001) and postoperative opioids (*p* = 0.012) may be at potential risk of publication bias. In contrast, female gender (*p* = 0.304), age (*p* = 0.089), non-smoking (*p* = 0.496), gynecologic surgery (*p* = 0.760), and laparoscopic surgery (*p* = 0.784) did not indicate significant publication bias.

## Discussion

This study revealed that the significant risk factors for PONV including patient-related (female, age, non-smoking, motion sickness, mmigraine, ASA), procedure-related (surgery duration, cholecystectomy, gynecology, breast, abdominal, fasting time), and anesthesia-related (anesthesia time, volatile-anesthetics, opioids, absence of ondansetron prophylaxis). The interpretation of the study results should be cautious due to the heterogeneity of the meta-analysis results. However, the NOS score, country, and study year as potential sources of heterogeneity were excluded through meta-regression analysis. The number of research centers was the only factor that could potentially cause heterogeneity in the age factor. This may be attributed to differences in population aging among different countries. There were many interventions available for PONV. However, PONV has never been effectively controlled. Even in patients who receive prophylactic antiemetic interventions, the incidence of PONV remains as high as 30–68% (51). The study results contribute to the systematic analysis of PONV-related risk factors, the construction of risk predictive models, the identification of high-risk patients, and the development of personalized prevention strategies.

The results of the combined analyses indicated that female gender was the strongest patient-related risk factor. Psychosocial factors, sex hormone levels, and other factors may contribute to a greater susceptibility to PONV in female patients. A recent study found that cyclical changes in female reproductive hormones may increase susceptibility to PONV (4, 52). Additionally, changes of female sex hormone levels may affect gastrointestinal transit time and the esophageal sphincter pressure (53). The risk of PONV would decrease with increasing age (54–56). However, this result was reversed in children, which may be attributed to the decline of autonomic reflexes. Because there are differences in neurological development between children and adults, this may affect the stability of the research results. Therefore, this study limited the research population to adults (>18 years). Motion sickness was an independent risk factor of PONV. A genome-wide association study indicated that patients with motion sickness were more likely to experience PONV (57). The results of meta-analysis showed that BMI was not a risk factor for PONV. Previous study suggested that elevated BMI would increase the risk of PONV since obese patients usually require higher doses of anesthetics and longer anesthetic duration, and is not simply attributable to the BMI itself (58). The mechanism by which smoking reduces the risk of PONV is currently unclear. Previous study reported that polycyclic aromatic hydrocarbons in tobacco smoke may promote the metabolism of anesthetics by inducing the production of cytochrome P450, thus reducing the risk of PONV (59, 60). Additionally, it has been suggested that the low susceptibility to PONV in smokers is due to changes in neurologic function as a result of nicotine withdrawal rather than nicotine exposure (10). The present study showed that migraine was an independent risk factor for PONV. However, the mechanisms involved are currently unclear. Previous studies have reported that the association of migraine with gastric disorders increases the risk of PONV (61, 62). Moreover, calcitonin gene-related peptide (CGRP) is associated with reduced gastric emptying (63). The *α* isoform of CGRP is associated with the pathogenesis of migraine, while the *β* isoform is associated with gastrointestinal motility (64). It has been hypothesized that this may explain the correlation between migraine and PONV. Additionally, the meta-analysis showed that a higher ASA classification may be a protective factor for PONV. This may differ from the general perception. This may be attributed to the fact that patients with a higher ASA classification are often older. Female patients experience a decrease in neurological sensitivity with age, which leads to a decrease in susceptibility to PONV. However, we must interpret these findings with caution. Statistical significance does not always equate to clinical relevance. For instance, the OR for BMI was 1.06, and for ASA II, it was 0.97. Although these associations were statistically significant, the effect sizes are close to unity. This suggests their clinical impact may be negligible compared to strong predictors like female gender (OR 2.39). Clinicians should prioritize factors with larger effect sizes for risk stratification.

Among the anesthesia-related factors, volatile anesthetics was the strongest risk factor. This may be related to the fact that volatile anesthetics were associated with a dose-dependent increase in the incidence of PONV (10). Meta-analysis showed that perioperative opioids increased susceptibility to PONV regardless of the time of administration. This is consistent with the results of previous studies (65). Opioids are associated with PONV by directly acting on *μ*-receptors in the central nervous system and gastrointestinal tract in a dose-dependent manner (66). N2O may contribute to PONV by inducing changes in middle ear pressure (67). However, this result was not significant in the present study. The results showed that ondansetron was a protective factor for PONV. However, the same finding was not significant in hormones. It is notable that the prophylactic effect of hormones for PONV may be altered depending on the dose of the drug and the population. Previous studies had indicated that the combination of hormones with other antiemetic drugs was more effective than single use (68, 69). Additionally, previous studies have shown that intravenous anesthesia and PCA were risk factors of PONV (8, 56, 70). However, this result was not significant in this meta-analysis. This may be attributed to the limitation of the number of included studies.

The present study found that there was an association between the duration of anesthesia and the duration of surgery and PONV. This is consistent with the results of previous studies (39, 71). This may be due to the fact that longer anesthesia times and surgery times may indicate more complex surgical procedures, surgical trauma, and consumption of anesthetic medications (e.g., opioids), thereby potentially increasing the risk of PONV. Additionally, some surgical procedures (e.g., cranial surgery, gallbladder surgery, gynecologic surgery, laparoscopic surgery) have been shown to be risk factors for PONV in previous studies (13, 51, 72). This is consistent with the results of this meta-analysis. The results of meta-analysis showed that cholecystectomy, gynecology, breast, and abdominal surgery were associated with an increased risk of PONV. Laparoscopic cholecystectomy was identified as a strong independent risk factor (OR 2.07). Given this high risk, targeted prophylactic interventions are crucial. A recent randomized triple-blind trial by Bauiomy et al. (2025) demonstrated that intraperitoneal administration of dexamethasone and dexmedetomidine significantly reduced PONV in these patients (73). This supports the implementation of procedure-specific multimodal protocols for high-risk surgeries. Notably, gynecologic surgery and breast surgery were associated with female gender factors, while gallbladder surgery, gynecologic surgery, abdominal surgery, and fasting time were associated with the digestive system. Motion sickness and postoperative opioid use may be subject to publication bias. This may lead to a tendency of reporting positive results.

### Implications

The significant risk factors in this study may be helpful in screening patients at high risk for PONV and improving existing risk prediction tools. The Apfel score is a widely used PONV assessment tool. This study supports the inclusion of specific factors in the Apfel score through systematic review and meta-analysis, and supplements other potential risk factors. Accurate risk stratification provides a basis for sustained PONV prevention strategies. Clinicians can quantitatively assign weights to different risk factors by referencing the OR values from this study, thereby improving the accuracy of risk stratification. Crucially, we distinguished between non-modifiable and modifiable risk factors to guide clinical decision-making. Non-modifiable factors include patient characteristics (e.g., female gender, motion sickness) and specific surgical procedures (e.g., cholecystectomy, gynecological surgery). These are vital for risk stratification to identify who needs prophylaxis. In contrast, modifiable factors, primarily anesthesia-related choices (e.g., volatile anesthetics, postoperative opioids), represent key targets for clinical intervention. To reduce PONV incidence, clinicians should prioritize Total Intravenous Anesthesia (TIVA) and opioid-sparing multimodal analgesia. This is particularly important for patients undergoing high-risk surgeries. Patients with multiple PONV risk factors require developing personalized multimodal interventions. Particularly for patients undergoing high-risk surgeries (e.g., cholecystectomy), avoiding volatile anesthetics and employing multiple antiemetics from different pharmacological classes can effectively interrupt distinct PONV pathways. Recent evidence reinforces this approach. For instance, Bauiomy et al. (2025) confirmed that combining varying pharmacological agents (e.g., intraperitoneal adjuvants) offers superior protection in high-risk laparoscopic procedures compared to monotherapy (73). Structured PONV prevention checklists ensure consistent application of prophylactic regimens. Recently, Singh et al. developed a continuous prevention protocol and real-world implementation checklist (74). This checklist mechanism helps bridge the gap between risk assessment and clinical action.

### Limitations

There were several limitations of this study. Only English-language literature was included in this study. This study summarized the adjusted odds ratios from multivariate regression models. However, primary studies did not control for all potential confounders. Unmeasured factors, such as preoperative anxiety or specific genetic markers, may still influence the results. Moreover, uncontrolled potential confounders (variations in antiemetic prophylaxis strategies and anesthesia protocols) may still introduce heterogeneity across the included studies. The differing sets of covariates across the various regression models summarized in this study may introduce ecological bias. The composite effect size represents an average association rather than a direct causal relationship. This study only assessed publication bias in meta-analyses with more than 10 included studies. Additionally, to ensure the robustness of regression estimates, studies with fewer than 600 patients were excluded in this study. However, this exclusion criterion does not preclude the possibility of neglecting data from smaller, specific patient cohorts. This study pooled data from both RCTs and observational cohorts. Although sensitivity analysis showed robust results, the methodological differences may introduce bias. Furthermore, the definitions of predictors varied across studies.

## Conclusion

This study revealed patient-related (female, age, non-smoking, motion sickness, migraine, ASA), procedure-related (surgery duration, cholecyst, gynecology, breast, abdominal), and anesthesia-related (anesthesia time, volatile-anesthetics, opioids, no ondansetron) significant risk factors for PONV. However, the interpretation of the study results should be cautious due to the heterogeneity of the meta-analysis results. The rigorously designed prospective study is required to confirm the findings of this study in the future.

## Data Availability

The original contributions presented in the study are included in the article/supplementary material, further inquiries can be directed to the corresponding author.
